# Progress in Diabetic Coma

**Published:** 1900-11-10

**Authors:** 


### PROGRESS IN DIABETIC COMA.

Recent researches on the pathology of diabetic coma have added very considerably to our knowledge of the causation of this formidable condition, and have led to the adoption of a method of treatment which has had a certain amount of success.

The resemblance of its symptoms to those of other forms of auto-intoxication by acids led to the suspicion that it is caused by the accumulation of some acetone in the system. Walter[1] pointed out that in diabetic coma, although ammonia in excess is present in the urine, it remains acid, and that since there is no diminution of other bases, which, indeed, increase in proportion to the amount of ammonia, there must be a great excess of some acid. The effect of an acid in excess in the blood would be to neutralise the normal alkalies, and seriously, or even fatally, interfere with the physiological functions of the organs.

Various organic acids have been suggested as the cause of diabetic coma, but the balance of probability is at present in favour of its being oxybutyric acid.

This is the opinion of Magnus Levy,[2] who has added materially to our knowledge of the pathology of this condition as the result of the careful study of eight cases.

As the neutralising power of oxybutyric acid is rather feeble, it must require the presence of a considerable quantity in the blood to produce serious toxic effects, and Levy calculated the amount at about 160 grammes for a male adult. Hitherto observers had failed to find in the urine a quantity of oxybutyric acid proportionate to that required to be in the blood; but Levy showed that this arose from defective methods, whereby the whole of the acid present had not been isolated.

As a logical sequence to the theory of acid auto-intoxication, treatment by a neutralising drug was employed, and there have been placed on record the results in 28 cases. Bicarbonate of soda was the salt employed, and in very large doses—up to 6 or 7 ounces daily. The results were only moderately satisfactory, as only seven recovered and seven improved under the treatment; but it must be borne in mind that previously the physician was helpless.

The cases of recovery, too, were those in which the treatment was begun early, and future statistics may be more encouraging.

Artificial serum, which has come into so general use on the Continent in septic conditions in enteric fever and in failure of the vital powers, has been tried in diabetic coma, and of eight cases recovery took place in two, whilst two showed improvement, treated by an artificial serum. Whilst the patient can swallow, the introduction of bicarbonate of soda can be pushed fully; but once he is profoundly comatose intravenous injection is the only available method. This reduces enormously the practicable dose, and it has been found that a litre and a half of a 3 per cent. solution of bicarbonate of soda is the extreme limit, this representing 45 grammes of the salt (about one ounce). Levy records a case of a diabetic youth, aged 13, who absorbed 200 grammes daily during two attacks, followed by recovery, and a patient of Besson, of Lille—in a state of profound unconsciousness, with imperceptible pulse and the movements of breathing as the only evidence of life—returned to complete consciousness an hour after an intravenous injection of 25 grammes of soda bicarbonate.

Citrate of soda is better tolerated than the bicarbonate, and it may be found that some other alkali gives better results, or some drug which stimulates elimination whilst diluting the blood.

Then, as regards preventive treatment, the appearance of any premonitory symptoms, or the presence of oxybutyric acid in the urine, should lead to the administration of alkalies, in doses corresponding to the urgency of the symptoms. The origin of the oxybutyric acid has not yet been made out, but it is probable that over-fatigue, whether mental or bodily, or a diet which fatigues the digestive and assimilatory organs, or is repugnant to the palate, tends to favour its production.

[1] Walter (Arch. für Experiment. Path. u Phar., vii. 2). [2] Magnus Levy (Arch. für Experiment. Path. u Phar., xlii. 2, 3, 4).

